# Thymosin α1 Interacts with Hyaluronic Acid Electrostatically by Its Terminal Sequence LKEKK

**DOI:** 10.3390/molecules22111843

**Published:** 2017-10-27

**Authors:** Walter Mandaliti, Ridvan Nepravishta, Francesca Pica, Paola Sinibaldi Vallebona, Enrico Garaci, Maurizio Paci

**Affiliations:** 1 Department of Chemical Sciences and Technologies, University of Rome “Tor Vergata”, via della Ricerca Scientifica 1, 00133 Rome, Italy; w.mandaliti@alice.it (W.M.); nepravishta@gmail.com (R.N.); 2 School of Pharmacy, East Anglia University, Norwich NR4 7TJ, UK; 3 Department of Experimental Medicine and Surgery, University of Rome “Tor Vergata”, via Montpellier 1, 00133 Rome, Italy; pica@uniroma2.it (F.P.); paola.sinibaldi@uniroma2.it (P.S.V.); 4 San Raffaele Pisana Scientific Institute for Research, Hospitalization and Health Care, 00163 Rome, Italy; garacienrico@gmail.com

**Keywords:** Thymosin α1, CD44, RHAMM, thymic hormone

## Abstract

Thymosin α1 (Tα1), is a peptidic hormone, whose immune regulatory properties have been demonstrated both in vitro and in vivo and approved in different countries for treatment of several viral infections and cancers. Tα1 assumes a conformation in negative membranes upon insertion into the phosphatidylserine exposure as found in several pathologies and in apoptosis. These findings are in agreement with the pleiotropy of Tα1, which targets both normal and tumor cells, interacting with multiple cellular components, and have generated renewed interest in the topic. Hyaluronan (HA) occurs ubiquitously in the extracellular matrix and on cell surfaces and has been related to a variety of diseases, and developmental and physiological processes. Proteins binding HA, among them CD44 and the Receptor for HA-mediated motility (RHAMM) receptors, mediate its biological effects. NMR spectroscopy indicated preliminarily that an interaction of Tα1 with HA occurs specifically around lysine residues of the sequence LKEKK of Tα1 and is suggestive of a possible interference of Tα1 in the binding of HA with CD44 and RHAMM. Further studies are needed to deepen these observations because Tα1 is known to potentiate the T-cell immunity and anti-tumor effect. The binding inhibitory activity of Tα1 on HA-CD44 or HA-RHAMM interactions can suppress both T-cell reactivity and tumor progression.

## 1. Introduction

Thymosin α1 (Tα1), a 28-amino acid polypeptide, is one of the peptides isolated for the first time from calf thymus extracts for which immune regulatory properties have been demonstrated both in vitro and in vivo [1,2,3]. It is acetylated on its N-terminus group and a sequence: Ac–Ser–Asp–Ala–Ala–Val–Asp–Thr–Ser–Ser–Glu–Ile–Thr–Thr–Lys–Asp–Leu–Lys–Glu–Lys–Lys–Glu–Val–Val–Glu–Glu–Ala–Glu–Asn–OH. Tα1 is cleaved by an asparagine endopeptidase (legumain, AEP) from the *N*-terminus of Prothymosin α [4]. Both Tα1 [4,5] and legumain [6] are present in different tissues, suggesting that this process is a general process in mammalian tissues.

Importantly Tα1 was recently proven to restore the functionality of the mutated form of the chlorine channel function in the cystic fibrosis [7], opening the way for a possible potent single molecule therapy of this pathology of cystic fibrosis.

Currently Tα1 is approved in different countries for the treatment of several viral infections [8,9,10,11] and as an adjuvant for immune enhancement [12,13]. Moreover, it has also been developed for treatment of non-small cell lung cancer (NSCLC), hepatocellular carcinoma, acquired immune deficience syndrome (AIDS) and malignant melanoma [14,15].

A univocal mechanism of action of Tα1 is still unknown since no specific receptors for Tα1 have been identified. Tα1 induces T-cell and dendritic cell (DC) maturation and interleukin (IL)-2 expression; it is also capable of upregulating the expression of some Toll-like receptors (TLRs) in murine DCs protecting mice from invasive aspergillosis in the MyD88 (myeloid differentiate factor 88)-dependent way [16,17,18,19,20,21,22,23,24]. Tα1 activates a protein kinase C (PKC)-IκB kinase (TRAF6) that is an atypical signaling pathway activating nuclear factor-κB and initiating the cytokine gene expression in murine bone marrow-derived macrophages [21].

Recent studies with model membranes indicate that Tα1, unstructured in water solution, assumes tracts of helical conformation in structuring solvents like trifluoroethanol [25,26]. Interestingly, it assumes a conformation with two helical domains and inserts the first 5 residues of the *N*-terminal helix [27,28,29,30] in negative membranes models and particularly upon the phosphatidylserine (PS) exposure as found in several pathologies and apoptosis [31].

These findings are in agreement with a growing body of evidence attesting the pleiotropy of this hormone peptide, which targets both normal and tumor cells interacting with multiple cellular components [16,17,18,19,20,21,22,23,24]; as such, they have generated a renewed interest. Studies of the serum Tα1 levels and of its interaction with soluble proteins [23,29] have also raised questions about the transport and localization of Tα1 in tissues and of the dynamics underlying the biological effects of this peptide in different clinical situations. All this research would benefit from a greater understanding of the Tα1 conformation and of the mechanism(s) involved in the interactions with other components of cells.

Hyaluronan (HA) [32] is a large, glycosaminoglycan containing repeating disaccharide units of *N*-acetyl glucosamine and glucuronic acid; it occurs ubiquitously in the extracellular matrix and on cell surfaces and is critical for maintaining their structure. While its structure is simple, HA is an extraordinarily versatile macromolecule. Its biosynthesis has been related to a variety of diseases as well as their developmental and physiological processes [32,33,34]. A number of cell-associated and extracellular HA binding proteins have been described as mediating the biological effects of HA [35], among them CD44 [36,37,38,39,40,41,42,43,44] and RHAMM [45,46,47]. Although information on the structure of these proteins is available, the specific requirements for their binding to HA is only available for CD44. HA can exist in high or low molecular weight forms due to cleavage into varying lengths. HA is considered the major ligand for CD44 and can bind CD44v isoforms that are ubiquitously expressed. Through binding of CD44, HA can activate cytoskeleton and matrix metalloproteinases signaling involved in tumor progression.

CD44 is a broadly distributed cell surface glycoprotein found on hematopoietic cells, fibroblasts, and numerous tumor cells and has seven extracellular domains, a transmembrane domain, and a cytoplasmic domain [42]. The CD44 variant v6 in particular promotes tumor progression and metastatic potential in some cancers. The extracellular structure contains clusters of conserved basic residues (BX7B motif) that are implicated in HA binding and located in the amino-terminal region in all isoforms [38,39,40,41,42,43,44].

RHAMM, the Receptor for HA-mediated motility, is a basic and coiled coil protein designated as CD168 and is located extra and intracellularly, including a HA-binding region with the same BX7B motif near its *C*-terminus [45,46,47].

HA may interact independently with both CD44 and RHAMM by common amino acid sequences [46]. It can induce numerous cell behaviors but specific inhibitors of these interactions are not available, thus the physiological functions of HA were deduced from the biological effects of some HA-receptor antagonists eventually blocking the interactions of HA with either CD44 or RHAMM [48,49,50,51]. Thus, it was hypothesized that it is likely that peptides able to bind HA can alter the HA-binding capacity of HA receptors [48]. Different HA-binding peptides have been proposed as specific, thus preventing leukocyte adhesion to HA and inhibiting leukocyte recruitment during contact hypersensitivity [51,52,53,54].

Thus, we carried out a preliminary investigation herewith reported about some points of similarity between the residues in the sequence of Tα1 and the common regions of CD44 and RHAMM able to bind HA [48,49,50,51,52,53]. Because a number of residues similar in their sequence were found, we considered the possibility that, at preliminary level, an interaction between Tα1 and HA can occur in these regions.

Thus, an investigation by NMR spectroscopy was carried out, making it possible to identify the modality of binding and the region of Tα1 where the interaction with HA occurs. Moreover, Tα1 is known to potentiate T-cell immunity and anti-tumor effect. The binding inhibitory activity of Tα1 on HA-CD44 or HA-RHAMM interactions can suppress both T-cell reactivity and tumor progression.

## 2. Results

### 2.1. The Comparison of the Aminoacidic Sequence between Thymosin α1 and the Common HA Binding Sites of RHAMM and CD44

**Figure 1:**
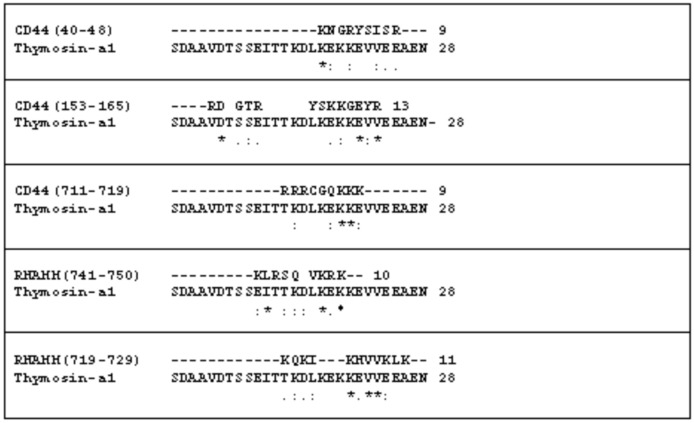
Sequence comparison between the residues of Thymosin α1 and the common hyaluronan binding motifs of both CD44 (Mouse) and RHAMM (Mouse) as reported [48]. The symbols (*), (**.**) and (**:**) indicate identity, similarity and high similarity respectively between individual residues.

The interactions of HA with RHAMM and CD44 occur by a common binding motif, indicated as BX_7_B, where B are Arg or Lys residues and X any amino acid with no basic character. Specifically, three and two different HA binding motifs were reported for CD44 and RHAMM respectively [48]. It is important to note initially that in the amino acid sequence of the Tα1 a clear BX_7_B motif is not present. Similarity between only some residues was found. In fact, in Figure 1 in the sequence comparison between Tα1 and HA common binding motifs of both RHAMM and CD44 proteins we report the residues that appear in a similar position. It is possible to note that comparing the regions 41–45, 153–162 and 711–719 of CD44 some other similarities are present. Some of similarities were also found in the alignment of Tα1 with 743–750 and 721–731 sequences of RHAMM.

It is important to note that the tracts (40–48, 153–165) of CD44 appear in a link module [55]. The tracts involved in RHAMM appear first in a loop (741–750) and second (719–729) in a helix. It is important to note that the structure proposed for RHAMM is formed by three long helices without a well-defined tertiary structure.

The common feature among the different comparisons of amino acid sequence analysis reported in Figure 1 is the presence of similarity and/or identity of some residues in the C-terminal region of Tα1, particularly where the lysine residues are located. It is important to note that probably these residue side chains with positive charges are able to form ionic bridges with HA negative charges.

### 2.2. 2D NMR Studies: Structural Characterization of Tα1 HA Interaction

**Figure 2:**
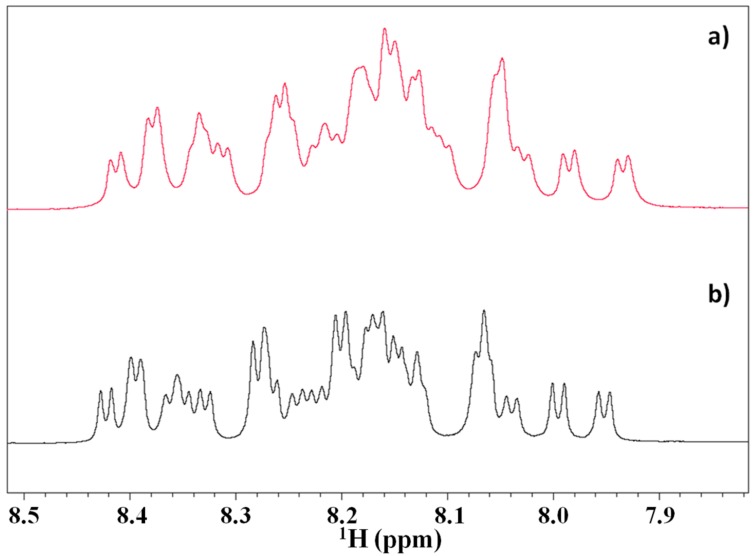
Amide protons region of the NMR spectra of (**a**) Tα1 in water; (**b**) Tα1 in presence of HA 0.4% (*w/w*).

The ^1^H NMR spectra of Tα1 (0.8 mM) in the presence of HA at the concentration of 0.4% (*w*/*w*) appear different with respect to the spectrum of the peptide in water solution. The differences observed in the amide region of the spectra, in particular the sharpening of the resonances in the presence of HA and the slight increase of the spectral dispersion, suggest that Tα1 interacts with HA, undergoing a partial structuration event removing the slow exchange between different conformers as in the case of a random coil conformation (Figure 2). On the other hand, a broadening of the resonances without any spectral dispersion should be interpreted as an aspecific binding between macromolecules.

**Figure 3:**
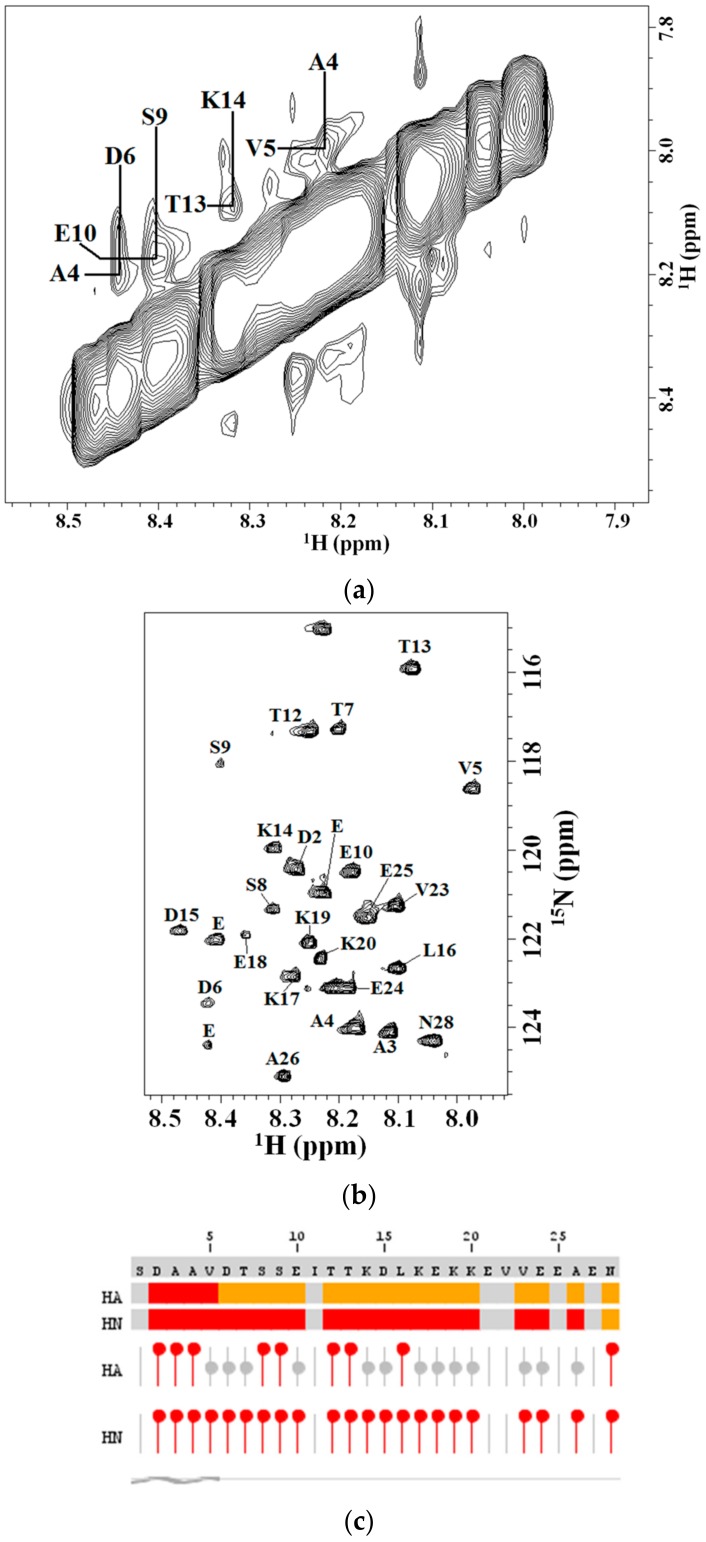
(**a**) NH–NH region of NOESY spectra of Tα1 in presence of HA 0.4% (*w*/*w*); (**b**) ^15^N-HSQC of Tα1 at natural abundance of rare isotope of Tα1 in presence of HA 0.4% (*w*/*w*) with individual assignments; (**c**) Chemical shift index (CSI) graphical representation obtained with the software NMRView according to Wright’s protocol [57] (see text). The algorithm predicts a helical conformation in the tract 1–5 of the peptide in presence of HA and an extended conformation in the rest of sequence.

The individual and sequential assignments of Tα1 were obtained by TOCSY and NOESY experiments with different mixing times as reported in the Materials and Methods. Heteronuclear 2D NMR spectra (^15^N-HSQC) at natural abundance (Figure 3a) were used to overcome ambiguities and complete the assignments of the homonuclear 2D NMR spectra using the previously reported results [27,28,29,30]. The appearance of NOEs in the NH–NH region of the NOESY spectra reported in Figure 3b allowed us to carry out the sequential assignment of the peptide; intense NH–NH (i, i + 1) NOEs strongly suggest the presence of a helical conformation [56].

After the complete assignments obtained on the basis of previous work on Tα1 [25,26,27,28,29,30] the preliminary analysis of the ^15^N chemical shift by the algorithm of Wishart and Sykes corrected for short peptides [57] confirmed values characteristic of the presence of a short tract in helical conformation from residue Ser1 to Val5 in presence HA at 0.4% (*w*/*w*) concentration (Figure 3c).

Thus, the 2D NMR assignments, the sequential NH-NH NOEs diagnostic of helix conformation [56] and the results of the chemical shift index protocol all revealed the existence of a tract helical conformation in the tract 1–5 of the peptide with some residues in the C-terminal with some helical propensity. The extended conformation of the rest of Tα1 is probably disordered, this being the best for the electrostatic binding to the periodic charges of HA as discussed below.

#### Diffusion NMR Studies

**Figure 4:**
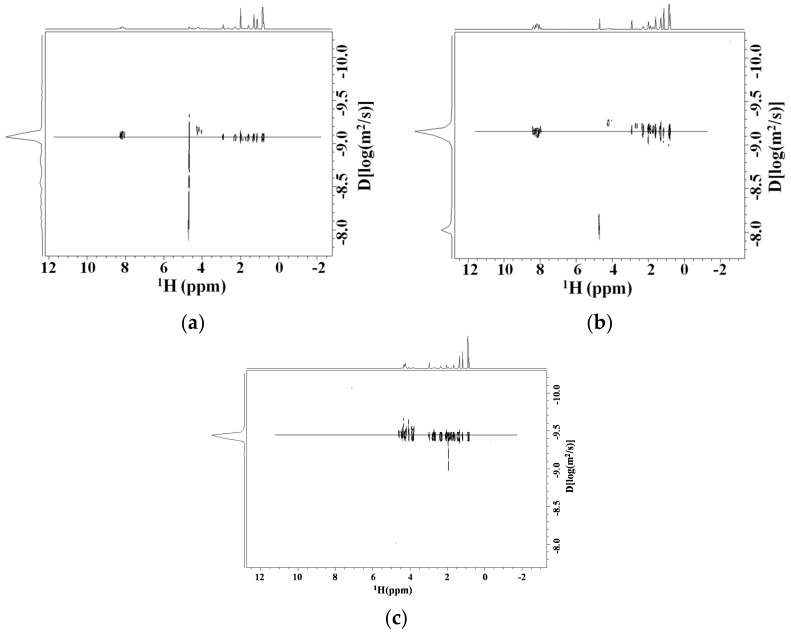
Molecular diffusion by NMR (DOSY spectra) of Tα1 in water solution (**a**) alone; (**b**) in presence of HA; (**c**) in the presence of the same concentration of dextransulfate.

The Diffusion NMR spectra (DOSY) of Tα1 (0.8 mM) in the presence of HA (0.4%, *w*/*w*) and at two different dilution steps (1:3 and 1:2) of the initial sample were acquired (see Figure 4). In the range of HA concentrations the marked differences of resonance intensity of the Tα1 at different dilutions are to be considered dependent on the change of the spin-spin relaxation time of peptide. This NMR parameter is dependent on the changes of the peptide’s tumbling, which can be directly attributed to the interaction of the peptide with the polysaccharide with a molecular weight higher than it. The measure at a different dilution but at the same molar ratio Tα1:HA resulted in a minimal change in diffusion, thus indicating that viscosity cannot be at the source of the value of the change of the diffusive front. This is to be attributed to the binding of Tα1 to the high molecular weight HA (See Figure 4). From the relationship between diffusion coefficients correlation times and molecular weight, it is possible to estimate that about 55% of total Tα1 concentration is bound to HA in the range of concentrations studied using the Stokes-Einstein model. This value accounts for a binding constant of Kd of Tα1 about 2.2 mM. This value is in line with an electrostatic interaction.

As a control the diffusion measure was performed in the presence in the same concentration of dextransulfate, a polyanion. The change of the diffusive front was clearly observed as reported in Figure 4c. In fact, the diffusion coefficient of Tα1 goes from the value of −9.08 in log(m^2^/s) units to −9.18 in the presence of HA to −9.41 in the presence of dextransulfate. This marked decrease in diffusivity should be due to the large electrostatic interaction of Tα1 with the negative charges of the polymer. It is important to remember that HA has alternating positive and negative charges every dimer. This is in line with the attribution to the interaction observed to an electrostatic interaction.

### 2.3. Magnetization Transfer by WaterLOGSY

**Figure 5:**
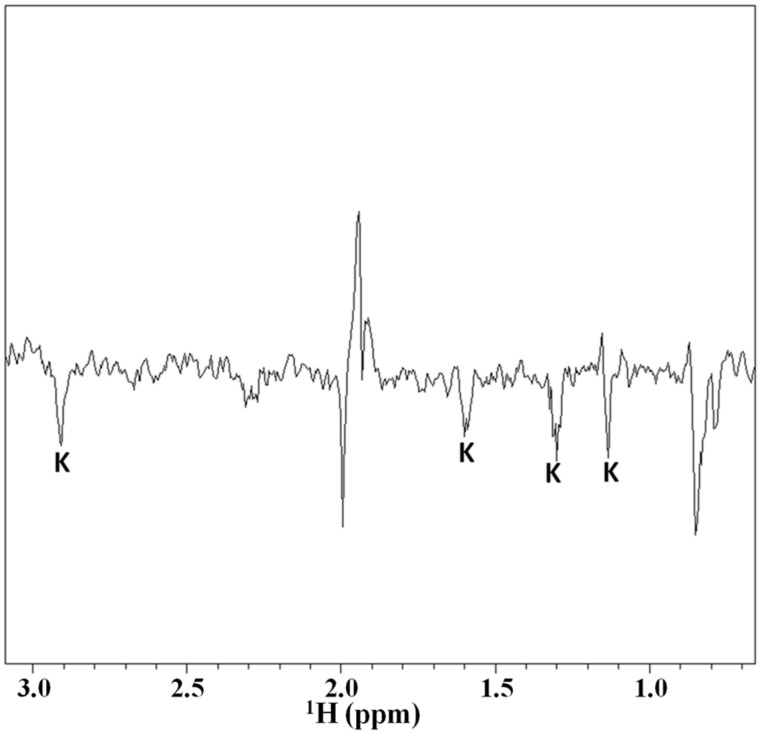
WaterLOGSY spectra obtained with Tα1 0.8 mM and HA 0.4% (*w*/*w*) H_2_O/D_2_O (90%/10%), the symbol K indicated the lysine proton resonances in the residue side chain.

In order to further investigate the interaction between Tα1 and HA the magnetization transfer by water saturation experiments were performed. The results of application of the WaterLOGSY method are reported in Figure 5. Particularly this NMR experiment makes it possible to obtain information about the specific residues of Tα1 involved in the interaction with HA.

**Figure 6:**
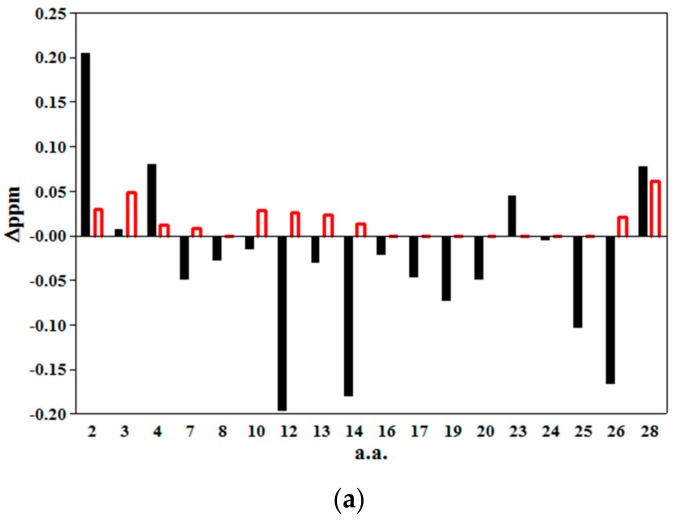

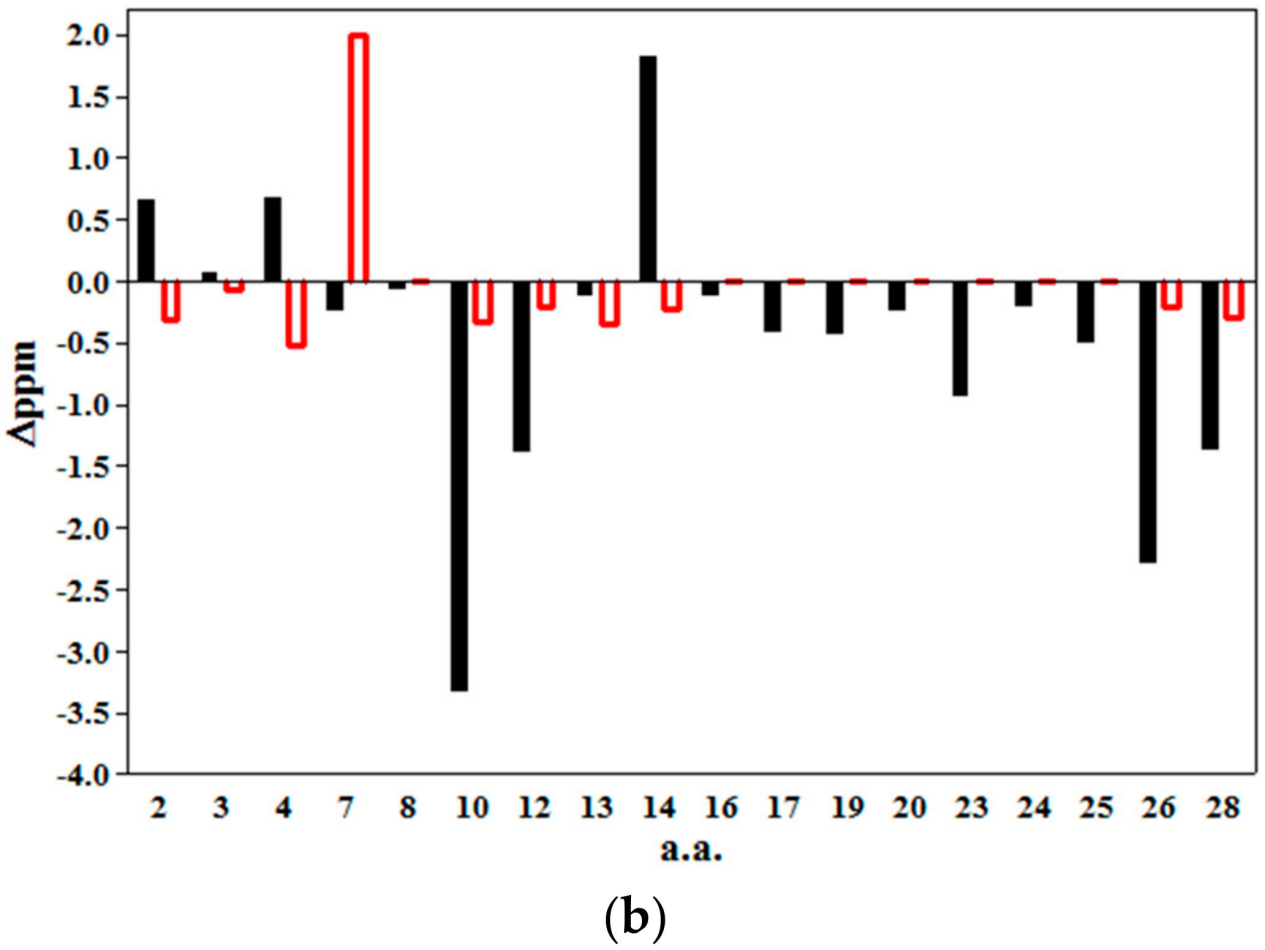
Bar graphs of Chemical shift difference between amide resonances of Tα1 in presence of PC-PS vesicles (black) and in presence of HA at a concentration of 0.4% (*w*/*w*) (red) (**a**); chemical shifts of amide proton and (**b**) chemical shifts of amide nitrogen.

In fact, in WaterLOGSY sequence the selective saturation of the water signal determines, in addition to others cross-relaxation pathways, a specific magnetization transfer between the water near to the binding site of a macromolecule (usually present in sub-stoichiometric amount) and the proton(s) of a certain ligand. Therefore, in the WaterLOGSY spectra are visible only the resonances due to protons of the ligand involved in the interaction with the macromolecule. The utility of this method is widely documented in literature in the studies of interaction between small molecules and macromolecules, such as DNA, RNA and proteins. The results of the application of the WaterLOGSY spectra reported in Figure 6 indicate that a magnetization transfer to all the resonances of the lysine side chains (ε, γ, δ, and β respectively) occurs, clearly suggesting that the lysine residues of Tα1 are directly involved in the interaction with the HA polymer.

### 2.4. Structuration Propensity of Thymosin α1: In Phospholipidic Vesicles and in Presence of Hyaluronic Acid

The chemical shift displacement of the amides resonances of Tα1, assigned in the natural abundance ^15^N-HSQC spectra of the rare isotope, in presence of phosphatidylcholine–phosphatidyserine (PC–PS) vesicles and in the presence of HA with respect to the same resonances of the peptide in glucose solution were monitored (spectra not shown). The bar graphs of the NH and the ^15^N displacements, see Figure 6a,b respectively, suggest that Tα1 in presence of HA shows a much smaller structuration effect in respect to the Tα1 in the presence of phospholipidic vesicles.

The graphs report the chemical shift perturbations [57,58] and confirm the slight structuration propensity only in the 1–5 tract of Tα1 in presence of HA with the remaining part remaining substantially in an extended conformation.

It is important to note that the “lysine region” (K14–K20) of Tα1 belonging to this region appears with a rather low structuration when it is involved in the interaction with HA, so confirming the results obtained by the WaterLOGSY.

It is important to remember that lysine residues are present in a tract with residues with similarity between Tα1 and the sequence able to bind HA of CD44 and RHAMM [46]. Overall the NMR studies gave a preliminary result that Tα1 interacts with HA by lysine residues, present in its C-terminal tract, accompanying a short helical structuration of the peptide’s tract 1–5.

## 3. Discussion

The results indicate that Tα1 has a similarity of sequence with the sequences common to both CD44 and RHAMM that interact with HA. These peptides have both been studied with different strategies [45,46,48] and by computer-aided design methods [59]. It was found that an octapeptide can bind to the HA binding domains of RHAMM. The docking experiments found that these peptides preferentially bound to the second helix forming three salt bridges between HA carboxylates and lysines side chains of RHAMM. Many results have been reported about these peptides together with some clusters of conserved basic residues (BX7B motif) [46,47,48,49].

Our approach started out from the sequence comparison between Tα1 with the common sequence found in the HA binding regions of CD44 and RHAMM [46,47,48,49]. The interaction of Tα1 with HA was investigated by NMR spectroscopy ^1^H and ^15^N HSQC and NMR diffusion studies. The results initially indicated that Tα1 interacts with HA. The NMR results confirm the direct involvement of the “lysine region” of the peptide in the interaction with hyaluronic acid, as suggested by WaterLOGSY NMR spectra. Upon binding, Tα1 assumes a very short helical structure in the *N*-terminus region while the remaining tract appears in an extended conformation including the region involved in the binding HA that contains the lysine residues. This is likely the region contains the LKEKK motif, which is the sequence also revealed in the sequence comparison above. 

Consistently, the synthetic peptide LKEKK corresponding to sequence 16–20 of human Tα1 and 131–135 of human interferon-α2 labeled with tritium binds with high affinity at nanomolar Kd T-lymphocytes [60]. Based on these findings it has been proposed that this fragment is responsible for Tα1 and IFN-α2 binding to various cells and that synthetic LKEKK peptide has similar binding capacity and exhibits biological activity.

The evidence of intermolecular interactions and their modalities presented in this paper, though absolutely preliminary, are a novelty in the field of Tα1. These studies are only indicative of a possible involvement of Tα1 in electrostatic interactions that may influence the HA binding with the specific receptors CD44 and RHAMM alone and in their complex interplay. Obviously, these results need to be validated by tests able to identify the biochemical pathways influencing the several activities of HA in modulating CD44 and RHAMM biological functions.

In fact, chemotherapeutic drugs and stimulatory cytokines are used to mobilize to the circulation hematopoietic progenitor cells in the blood, but the mechanisms are largely unknown. Mobilization is almost certainly an active process which involves an initial step modulating adhesion receptors to permit detachment and a second step in which motile behavior is stimulated to permit migration [61,62]. Hyaluronan can play a key role in structuring tissue architecture, and is an important component in motility of normal and malignant hematopoietic cells, including T-cells, B cells, monocytes, and thymocytes. CD44, a receptor for HA, has been shown to participate in the adhesion of normal and malignant stem cells to the extracellular matrix.

RHAMM, another receptor for HA mediated motility, regulates cell cycling, transduces signals, and dissolves focal adhesions. In contrast to CD44, RHAMM mediates motility, or de-adhesion of all hematopoietic cells tested to date; this suggests that RHAMM interactions with HA may facilitate migratory behavior, whereas CD44 interactions with HA may facilitate anchoring [61,62].

It is interesting to note that in some reports Tα1 somehow influences most of these processes. In fact, Tα1 has been shown capable of increasing lymphocytic infiltration to sites of disease [63,64,65,66,67] while a correlation between tumor-infiltrating lymphocytes and prognosis for patients with stage IV cancer has been documented [68]. Moreover, Tα1 can reduce apoptosis of immune cells, as shown in mouse [69,70] and human [71,72] thymocytes, and increase stem cell expansion in immunosuppressed mice [73,74,75].

These findings explain the therapeutic efficacy of Tα1 in several types of cancer, especially in combination therapy protocols [64], based on its dual action on immune effector and tumor cells. The increase in effectiveness of chemotherapy has been linked to the Tα1-induced increase in tumor infiltrating lymphocytes, upregulation of antitumor T-cells and enhanced expression of cell-surface and tumor markers, with a consequent increased tumor cell immunogenicity. The decrease in side effects of chemotherapy has been linked to the Tα1 immuno-restorative properties, such as its ability to induce maturation, differentiation and function of immune effector cells and to increase regulatory T-cells with consequent decreases in pro-inflammatory cytokines [75].

The above biological actions may also contribute to explaining the therapeutic efficacy of Tα1 in other physio-pathological conditions associated with immunological dampening and persistent inflammation, such as chronic viral hepatitis, severe sepsis, primary or acquired immuno-deficiencies and aging [13].

In this scenario, new questions arise. Further research on the interaction of Tα1 with cell surface receptors and key extracellular matrix components is needed for a better understanding of the mechanisms involved in the multiple biological effects of this peptide on immune response, inflammation, cancer and chronic hepatitis, also in relation to the remodeling of tumor microenvironment and the mechanisms involved in tissue repair [75]. In this regard the interaction of Tα1 with HA is highly intriguing, considering the ability of hyaluronan to act as a ligand for some receptors on cell membranes and to control cell cycle and tumor progression. In fact, cell-based experiments liking pre-incubation of Tα1 with HA on T-cell responses (such as cell proliferation, cytokine production … etc.) in comparison to HA-treated or Tα1 -treated T-cell alone may give important information. These preliminary results need, however, to be confirmed, substantiated and verified by a complex work in cell cultures by the laboratory experiments beyond the study in vitro.

## 4. Materials and Methods

The authors declare that the research did not involve Human Participants and/or Animals.

Thymosin α1 was a generous gift of SciClone Pharmaceuticals, Inc. (San Mateo, CA, USA). Sodium hyaluronate “High MW” with a value about MW ≥ 500 kDa for pharmaceutical and medical use was purchased from “htl”, Javené, France.

### 4.1. Sequence Homology

The alignments between Thymosin α1 and different regions of CD44 and RHAMM proteins were performed using ClustalOmega program, available in the UniProt web site [76].

### 4.2. Preparation of Samples for NMR Measurements

For two-dimensional (2D) NMR and WaterLOGSYspectra the sample contained Tα1 and HYA at a concentration of 0.8 mM and 0.4% (*w*/*w*) respectively, with D_2_O at 7% for lock signal. The DOSY NMR spectra were acquired on the same sample and at two different dilutions steps, of 1:3 and 1:2. The control with dextran sulfate was obtained by mixing Tα1 with dextransulfate (Pharmacia, Uppsala, Sweden). The pH of all the solutions was adjusted to 6.5, and then the samples were placed into 5 mm NMR tubes for data collection at 298 K using sodium trimethylsilyl propionate (TSP) for internal reference.

### 4.3. NMR Spectroscopy

NMR spectra of Tα1 alone and in the presence of HA were run at 298 K on a Bruker Avance instrument operating at 700.13 MHz. ^1^H NMR spectra were performed with both the zgpr and zgpesgp pulse program of the Bruker library for water signal suppression, usually with 32 scans and a relaxation delay of 2 s. Two-dimensional NMR experiments were performed in phase sensitive mode with a time proportional phase increment (States−TPPI) cycle [77] typically using 2 K of memory for 512 increments. The number of scans was optimized to obtain a satisfactory signal-to-noise ratio. Correlation experiments were performed with a TOCSY pulse sequence with the MLEV-17 spin-lock composite pulse sequence inserted [78,79] with mixing times of 60, 150, and 200 ms. NOESY spectra were obtained using the classical pulse sequence [56,80] with mixing times from 0.20 to 0.30 s. A sine bell apodization function shifted typically by π/2 in both dimensions was applied. In all homonuclear 2D experiments, a 1024 × 1024 matrix in phase sensitive mode was thus obtained with a digital resolution of ~5 Hz/point. ^1^H-^15^N-HSQC [81] natural abundance spectra were acquired to confirm the assignments and to monitor the changes in Tα1 amide groups in the different cases described (see Results). The TOPSPIN 3.1 and NMRView software packages were used for data processing and analysis. The Chemical Shift Index [56] calculations were performed using Wright’s protocol incorporated into NMRView. The binding of Tα1 to HA was investigated by a diffusion-ordered spectroscopy (DOSY) method [82,83] and by WaterLOGSY NMR methods [84,85]. The DOSY spectra were recorded on a Bruker Avance instrument operating at 700.13 MHz by using the ledbpgppr2s pulse sequence to suppress the water signal. During the DOSY experiment, 32 monodimensional spectra were acquired with 64 scans in a linear increasing gradient varying from 5 to 95% with a Δ of 70 ms and a δ of 2 ms. The spectra were then analyzed using the DOSY module implemented in Bruker software TOPSPIN 3.1. The WaterLOGSY measurements were performed on a Bruker Avance instrument operating at 700.13 MHz by using the pulse sequence proposed by Dalvit [84,85] incorporating the Excitation Sculpting pulse scheme with gradients for water suppression [86]. The WaterLOGSY spectrum collected was the sum of 1024 scans.
